# Carapace microbiota in American lobsters (*Homarus americanus*) associated with epizootic shell disease and the green gland

**DOI:** 10.3389/fmicb.2023.1093312

**Published:** 2023-04-05

**Authors:** Anna Schaubeck, Dianjun Cao, Vincent Cavaleri, Seyoung Mun, Soo Jin Jeon

**Affiliations:** ^1^Department of Veterinary Biomedical Sciences, College of Veterinary Medicine, Long Island University, Brookville, NY, United States; ^2^Division of Marine Resources, New York State Department of Environmental Conservation, East Setauket, NY, United States; ^3^Center for Bio Medical Engineering Core Facility, Dankook University, Cheonan, Republic of Korea; ^4^Department of Microbiology, College of Science and Technology, Dankook University, Cheonan, Republic of Korea

**Keywords:** American lobsters, epizootic shell disease, *Aquimarina*, *Tenacibaculum*, carapace microbiota, green gland, *Halocynthiibacter*

## Abstract

Epizootic Shell Disease (ESD) has posed a great threat, both ecologically and economically, to the American lobster population of Long Island Sound since its emergence in the late 1990s. Because of the polymicrobial nature of carapace infections, causative agents for ESD remain unclear. In this study, we aimed to identify carapace microbiota associated with ESD and its potential impact on the microbiota of internal organs (green gland, hepatopancreas, intestine, and testis) using high-throughput 16S rRNA gene sequencing. We found that lobsters with ESD harbored specific carapace microbiota characterized by high abundance of *Aquimarina,* which was significantly different from healthy lobsters. PICRUSt analysis showed that metabolic pathways such as amino acid metabolism were enriched in the carapace microbiota of lobsters with ESD. *Aquimarina*, *Halocynthiibacter,* and *Tenacibaculum* were identified as core carapace bacteria associated with ESD. Particularly, *Aquimarina* and *Halocynthiibacter* were detected in the green gland, hepatopancreas, and testis of lobsters with ESD, but were absent from all internal organs tested in healthy lobsters. Hierarchical clustering analysis revealed that the carapace microbiota of lobsters with ESD was closely related to the green gland microbiota, whereas the carapace microbiota of healthy lobsters was more similar to the testis microbiota. Taken together, our findings suggest that ESD is associated with alterations in the structure and function of carapace microbiota, which may facilitate the invasion of bacteria into the green gland.

## Introduction

The American lobster (*Homarus americanus*) is the most economically valuable species in the US fishing industry, distributed along the western North Atlantic coastal waters. Long Island Sound (LIS), a large estuary located between the Connecticut and Long Island shores, was one of the largest lobster habitats in the US until the late 1990s (Long Island Sound Study; National Oceanic and Atmospheric Administration). However, lobster populations have dramatically declined in the region over the past two decades (Long Island Sound Study; National Oceanic and Atmospheric Administration). It is believed that warming water temperature and resultant disease are the key factors driving the dramatic decline in lobster populations (Howell et al., 2005; Howell, 2012; Shields, 2013; Groner et al., 2018). The average temperature of the LIS bottom water has risen a total of 1.6°C (0.4°C per decade) since 1976 (Groner et al., 2018). The elevated temperature not only limits survival and reproduction of lobsters, but it can also make them relocate from their habitats (National Oceanic and Atmospheric Administration, 2016; Quinn, 2017; Goldstein et al., 2022). In addition, increasing ocean temperature enables opportunistic marine pathogens to proliferate and increase their virulence, leading to disease emergence such as Epizootic Shell Disease (ESD; Harvell et al., 2002; Tlusty and Metzler, 2012; Shields, 2013, 2019). Indeed, high prevalence of ESD has been reported in LIS lobster stock, which corresponds to a decline in lobster abundance (Castro et al., 2012; Groner et al., 2018).

ESD is characterized by pitting, melanization, and erosion of the carapace of the American lobster, which can be classified into three categories based on the extent of bacterial invasion and cuticular erosion (Smolowitz et al., 2005): Category 1 symptoms are the least severe, with little to no inflammation and shallow lesions, as well as small areas of melanization on the cuticle. Category 2 symptoms present as deeper lesions, degradation of the proteins within the chitin lattice formation, and indications of an immune response by the presence of moderate numbers of hemocytes. Category 3 symptoms are the most severe, with carapace ulceration in which the cuticle is completely absent, severe melanization of exposed portions, and highly inflamed tissue covering the pseudomembrane. Lobsters with ESD reduce the value of commodities and even progress to death. Nonetheless, the etiological agents for ESD have not been completely identified.

It has been proven that ESD is associated with dysbiotic shift in the carapace bacterial community (Bell et al., 2012; Chistoserdov et al., 2012; Meres et al., 2012). This means that ESD may not only be caused by a single species, but by polymicrobial infections. However, there is little information about carapace microbiota associated with ESD, and it is unclear if they can access internal organs through the cuticles. This study aims to characterize the structure and function of carapace microbiota of American lobsters with ESD and determine similarities between microbiota of the hepatopancreas, intestine, green gland, and testis in the same individuals using 16S rRNA gene sequencing. This study will contribute to development of management strategies for rebuilding and conservation of lobster populations by improving our understanding of ESD.

## Materials and methods

### Sampling of lobsters

**Figure 1:**
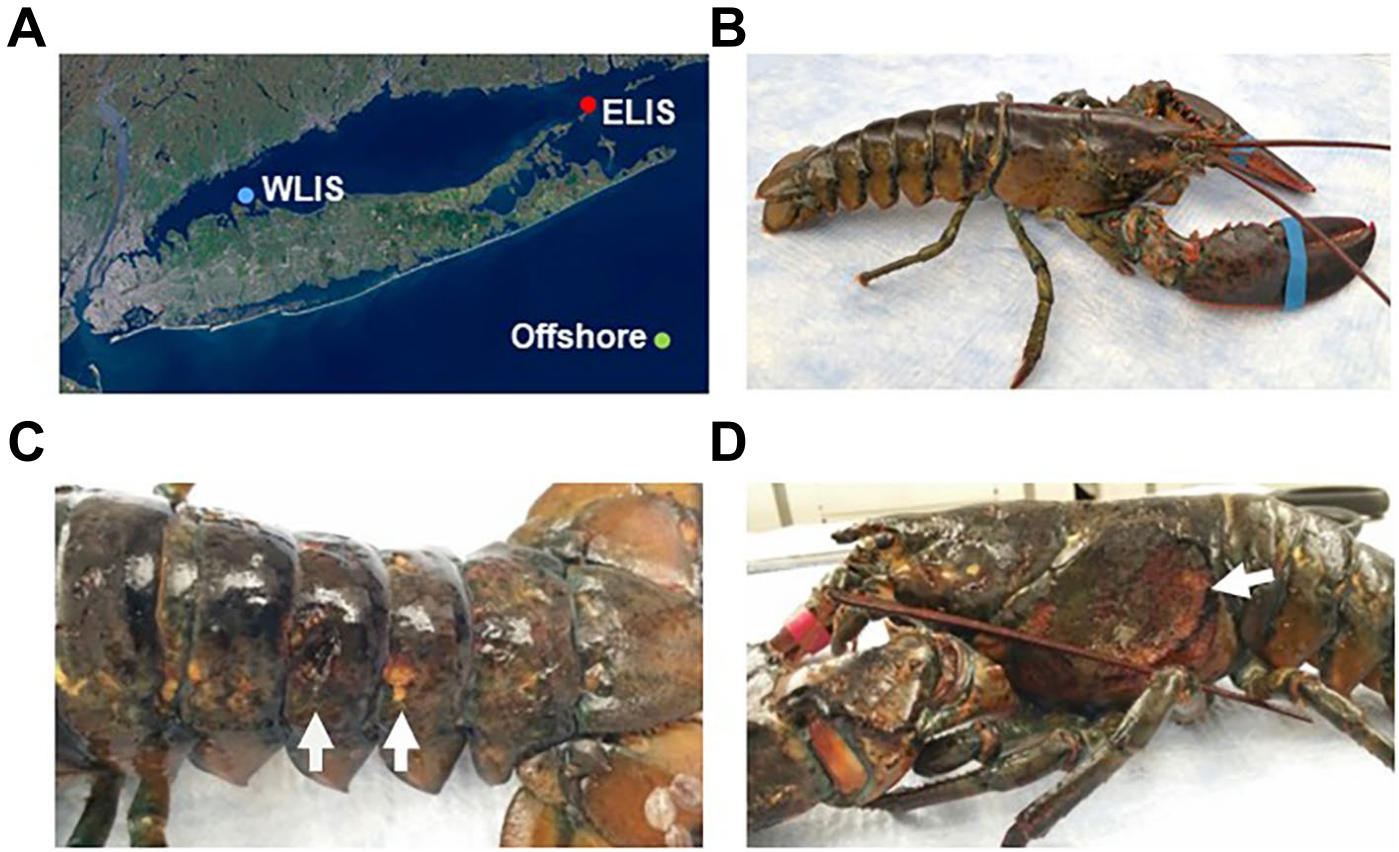
Collection of American lobsters from Long Island Sound. **(A)** Sampling locations: Western Long Island Sound (WLIS), Eastern Long Island Sound (ELIS), and 50 miles south of Montauk (offshore). Lobsters with ESD were only collected from ELIS and offshore. **(B)** Healthy lobster. **(C)** Lobsters with ESD showing pits and erosion. **(D)** Lobsters with ESD showing an ulceration.

In support of local lobstermen and the New York State Department of Environmental Conservation, American lobsters were collected from WLIS in July 2019, 50 miles south of Montauk in October 2019, and ELIS in August 2020 (Figure 1A; Supplementary Table S1). The lobsters were immediately placed in a cooler with ice packs to keep them alive and delivered to the lab within 2 h. Healthy and diseased lobsters were kept separately to avoid cross-contamination. Weight and length measurements were taken, and the lobsters were then dissected to collect various tissues, such as the carapace, green gland, hepatopancreas, intestine, and testis. Following a gentle wash with phosphate-buffered saline (PBS) at pH 7.4, the samples were stored in RNA*later* stabilization solution (Qiagen) at −80°C until microbial genomic DNA extraction. The length from the rear of the eye socket to the end of the carapace was an average of 25.4 cm (23.5–27.0 cm) and the weight an average of 632.4 g (528.5–763.5 g). Lobsters with shell lesions regardless of severity were classified as diseased (ESD, *n* = 8), and lobsters with no apparent signs of carapace lesions were considered healthy (HTH, *n* = 10). Metadata for sample information is available in Supplementary Table S1.

### Microbial genomic DNA extraction

Microbial genomic DNA was extracted from both shells and internal organs of lobsters using the DNeasy Blood & Tissue kit (Qiagen) according to the manufacturer’s protocols, with a minor adjustment in the pretreatment of the shells. Briefly, shells in RNA*later* stabilization solution were cut into small square pieces, placed in a microcentrifuge tube in 320 μl of PBS, and incubated on ice for 1 h to help detach bacteria from the shells. The incubated shells in PBS were disrupted and homogenized using a handheld homogenizer (Bel-Art). The resulting homogenizing solution, which contains bacteria from the surface of shells as well as bacteria present in the shells, was subsequently used for DNA extraction. For internal organs, 10–25 mg of the hepatopancreas, intestine, green gland, and testis were used for the extraction of DNA according to the manufacturer’s instructions. The DNA concentration and purity from all samples were measured by a NanoDrop One^C^ spectrophotometer (Thermo Scientific).

### Library preparation and illumina MiSeq sequencing

16S rRNA V3-V4 amplicon sequencing library preparation and Illumina MiSeq sequencing were conducted at GENEWIZ, Inc (South Plainfield, NJ, United States). Briefly, microbial genomic DNA for each sample was normalized to 20 ng/μl using a Qubit 2.0 Fluorometer (Invitrogen) and was used to generate amplicons using a MetaVx™ Library Preparation kit (GENEWIZ). The V3-V4 region of the 16S rRNA gene was amplified using the forward and reverse primers (341 Forward: CCTACGGRRBGCASCAGKVRVGAAT, 806 Reverse: GGACTACNVGGGTWTCTAATCC). Second limited-cycle amplification was performed to add multiplexing indices (barcode) and Illumina sequencing adapters. DNA libraries were validated by Agilent 2100 Bioanalyzer (Agilent Technologies) and quantified by Qubit 2.0 Fluorometer (Invitrogen). DNA libraries were multiplexed and loaded on an Illumina MiSeq instrument according to manufacturer’s instructions (Illumina). Sequencing was performed using the 2×250 paired-end configuration, and image analysis and base calling were conducted by the MiSeq Control Software embedded in the Illumina MiSeq instrument.

### Sequence data analysis

Two carapace samples from ESD and one from HTH were excluded from the analysis due to sequencing failure. Detailed information on the sequencing data statistics and quality is provided in Supplementary Table S2. 16S rRNA data analysis was completed with the QIIME2 package (2022.v8) in Python where forward and reverse reads were joined and assigned to samples based on their barcodes (Bolyen et al., 2019). After removing the barcode and primer sequences using qiime2/q2-cutadapt, quality filtering on joined sequences was performed. Sequences were discarded if they did not meet the following the parameters: Sequence length > 200 bp, min quality = PHRED 4, quality-window = 3, and min length fraction = 0.75. Sequences that passed this quality filtering were used to perform the next bioinformatics analysis. The QIIME package was used to perform operational taxonomic units (OTUs) picking, and the sequences were clustered into OTUs using the VSEARCH plugin with a clustering threshold of 99% sequence identity against the pre-clustered Silva 138 database (min merge length = 250; Rognes et al., 2016). A taxonomic category was assigned to each OTU with a confidence threshold of 0.7 using the Ribosomal Database Program (RDP) classifier. To standardize the OTUs data matrix across all samples produced by 16S rRNA gene sequencing, rarefaction curves were calculated with the satisfied depth (>155) using the QIIME (v1.9.1.) alpha-rarefaction curve analysis package. For the α-diversity analysis, diversity indices (ACE, Chao1, Shannon, and Simpson) were computed for each sample and compared between ESD and HTH using the QIIME plug-in. To assess similarities and differences between the carapace microbiota of ESD and HTH at the genus level, a principal coordinate analysis (PCoA) based on Bray-Curtis, weighted UniFrac, and unweighted UniFrac distances was conducted and visualized using ggplot2 in the R package v4.1.3 (Wickham, 2016). PCoA plots for microbiota obtained from the green gland, hepatopancreas, intestine, and testis were performed using Bray-Curtis distance and plotted using PAST3 (v4.03; Hammer et al., 2001). Venn diagrams showing the number of core genera shared by all lobsters within each group, the number of bacterial genera with an average relative abundance of ≥0.1%, and the number of bacterial genera present in any of the samples from each group were created using Bioinformatics & Evolutionary Genomics.^1^ We plotted the 10 most abundant bacterial genera within each body site as bar graphs or stacked bar graphs using GraphPad Prism v8.4.2 (GraphPad Software, San Diego, United States). Functional profiles of carapace microbiota were predicted based on 16S rRNA genes using Phylogenetic Investigation of Communities by Reconstruction of Unobserved States (PICRUSt) v1.1.4 (Langille et al., 2013), and were analyzed in the Statistical Analysis of Metagenomic Profiles (STAMP) v2.1.3 (Parks et al., 2014) to identify and visualize functional genes (level 3 KEGG orthology) that were statistically significant between groups. Hierarchical clustering analysis of five tissues based on the genus abundance data was conducted using the hclust function with the ward.D2 method in the R package v4.2.2 (Gregory et al., 2021).

### Statistical analysis

To compare carapace microbiota between groups, we conducted a non-parametric permutational multivariate analysis of variance (PERMANOVA) test on the Bray-Curtis, weighted UniFrac, and unweighted UniFrac distances using pairwiseAdonis (v0.4) in the R package (v4.1.3). We also analyzed the similarity of microbiota obtained from the green gland, hepatopancreas, intestine, and testis between ESD and HTH within each body site using PERMANOVA on the Bray-Curtis distance in PAST3 (v4.03). The relative abundance of bacterial taxa was reported as means with standard errors of the mean (SEM), and differences between ESD and HTH were assessed using the Mann–Whitney U test in the R package (v4.1.3). The abundance of KEGG orthology gene families between groups was analyzed using the two-sided, non-parametric White’s *t*-test in STAMP (v2.1.3). We considered differences with a *p*-value ≤0.05 as statistically significant.

## Results

### Sampling location and clinical symptom

American lobsters were obtained from three regions (Figure 1A): Eastern Long Island Sound (ELIS), Western Long Island Sound (WLIS), and 50 miles south of Montauk. Lobsters with ESD were only found in ELIS and offshore. Healthy lobsters showed no apparent signs of lesions on the carapace (Figure 1B), while lobsters with ESD mostly showed pits or erosion over the entire body (Figure 1C). There was one severe case with ulcers that removed the carapace and exposed underlying connective tissues to the external environment (Figure 1D). This may show the possibility that carapace bacteria can penetrate to the internal organs through shell lesions.

### Results from Illumina sequencing

Forty samples were sequenced on the Illumina MiSeq, generating an average of 54,118 high-quality reads per sample with >Q20 (97%) and > Q30 (94%). The average number of non-chimeric reads filtered through the QIIME2 pipeline was 17,384, which were used to cluster bacterial OTUs through the SILVA 138v 16S rRNA gene reference database with a 70% classification confidence threshold. A total of 4,954 OTU classifications were generated, of which 1,022 were classified at the genus level across 40 samples. The OTU classification data also provided a framework for understanding microbial distribution and diversity by lobster tissue or ESD (Supplementary Table S2).

### Diversity of carapace microbiota

**Figure 2:**
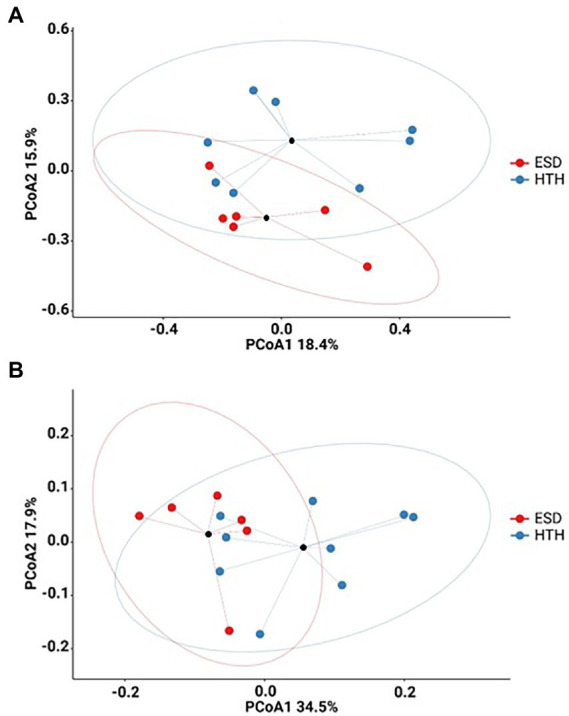
Carapace microbiota associated with ESD. **(A)** Bray-Curtis PCoA plot (PERMANOVA, *p* = 0.018). **(B)** Weighted UniFrac PCoA plot (PERMANOVA, *p* = 0.024). Carapace microbiota from ESD (red) was significantly different from HTH (blue). The black circles represent the mean values, and ellipses indicate 95% confidence intervals.

To determine species diversity in a sample, we examined the α-diversity indices including the ACE, Chao1, Shannon, and Simpson (Supplementary Figure S1). The ESD group showed 150.98 ± 66.09 for ACE, 141.15 ± 65.64 for Chao1, 4.91 ± 0.55 for Shannon, and 0.93 ± 0.03 for Simpson. The HTH group presented 227.20 ± 106.73 for ACE, 211.51 ± 101.94 for Chao1, 5.27 ± 0.90 for Shannon, and 0.94 ± 0.05 for Simpson. There were no significant differences (Mann–Whitney U test) in α-diversity indices between ESD and HTH. To determine the similarity and dissimilarity of carapace microbiota between the ESD and HTH groups, we performed the β-diversity analyses at the genus level using PCoA based on the Bray-Curtis (Figure 2A) and weighted UniFrac (Figure 2B) distances. The first two axes of Bray-Curtis PCoA explained 18.4 and 15.9% of variation in bacterial community structure, respectively, with significant separation of carapace microbiota between ESD and HTH (PERMANOVA, *p* = 0.018). The first two axes of weighted UniFrac PCoA explained 34.5 and 17.9% of variation in bacterial community structure, respectively, with significant separation of carapace microbiota between ESD and HTH (PERMANOVA, *p* = 0.024). Meanwhile, unweighted UniFrac PCoA showed no significant separation (PERMANOVA, *p* = 0.18) between ESD and HTH (Supplementary Figure S2). The data suggest that changes in relative abundances of microbial taxa are likely attributed to distinct carapace microbiota in ESD.

### Carapace microbiota structure and function associated with epizootic shell disease

**Figure 3:**
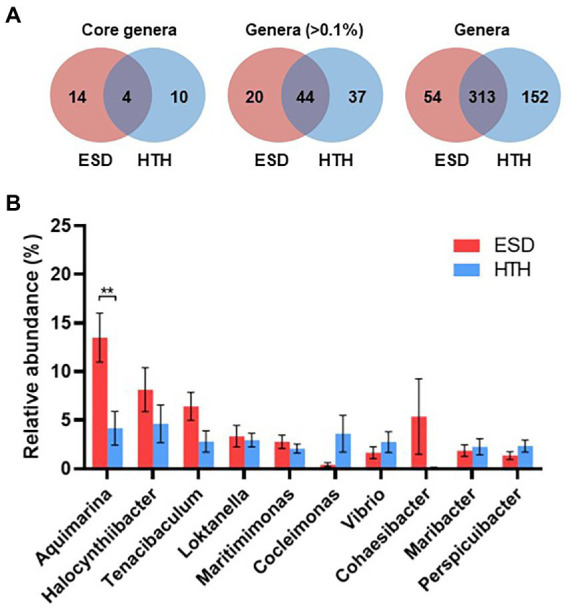
Structure of carapace microbiota at the genus level. **(A)** Venn diagrams showing the number of core bacterial genera shared by all lobsters within each group regardless of their abundance, the number of bacterial genera with >0.1% abundance from any of the samples within groups, and the number of bacterial genera found in any of samples within groups. **(B)** Relative abundance of the 10 most abundant genera at >1% abundance in all carapace samples. The bars represent the means and their standard errors, and asterisks indicate a statistical significance between groups (Mann–Whitney U test, **p < 0.01).

**Figure 4:**
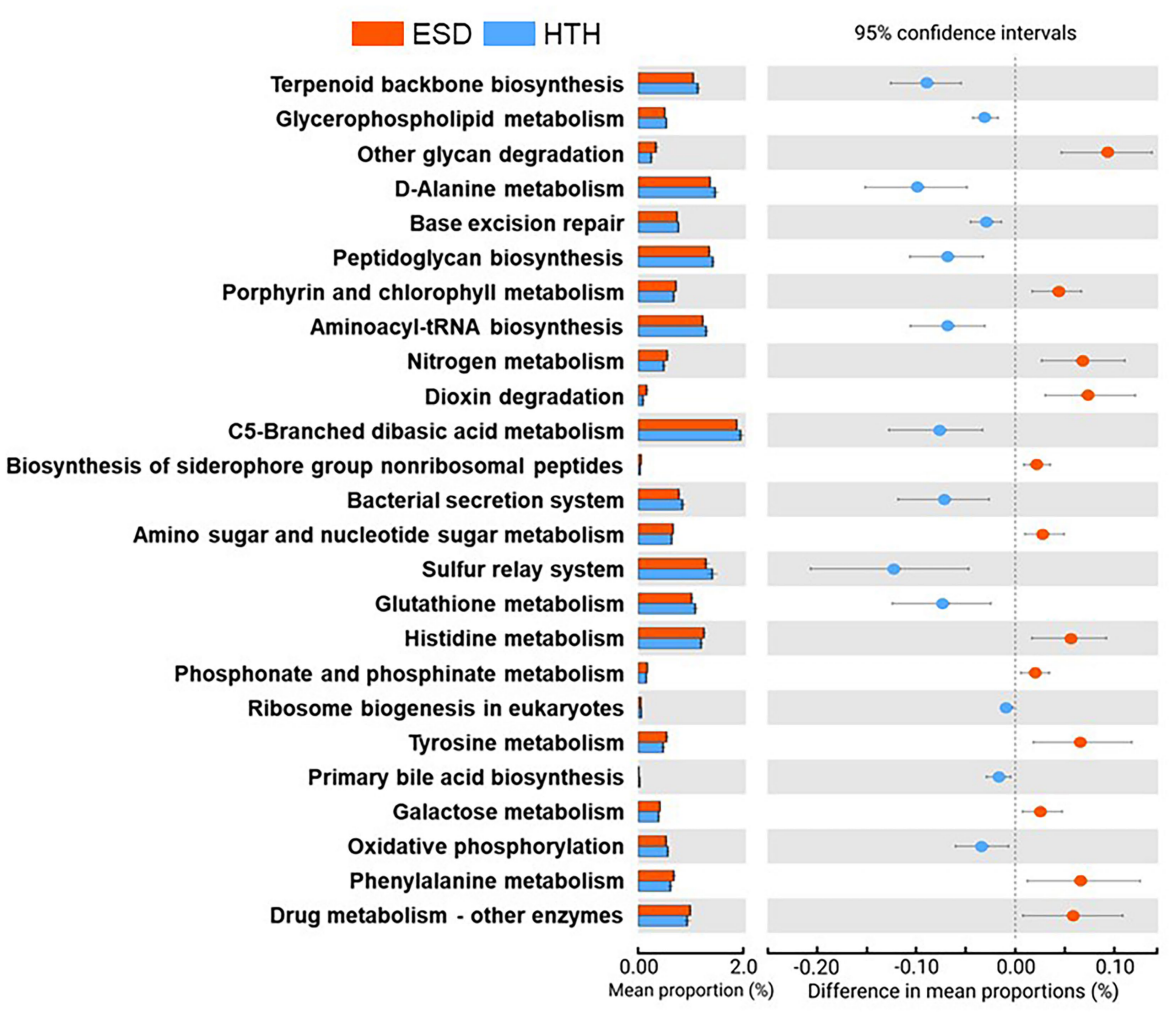
Functions of carapace microbiota. Extended error bar plot indicates 25 KEGG orthology gene families that were significantly different between ESD and HTH (White’s non-parametric *t*-test, two-sided, *p* ≤ 0.05).

We analyzed and compared the carapace microbiota structure between lobsters with ESD and healthy lobsters. Lobsters with ESD had a significantly higher abundance (*p* = 0.04) of the phylum *Bacteroidota* (formerly *Bacteroidetes*) in their carapace microbiota, whereas healthy lobsters exhibited a higher abundance (*p* = 0.05) of the phylum *Pseudomonadota* (formerly *Proteobacteria*). Detailed information on the relative abundance of bacteria at each taxonomic level is provided in Supplementary Table S3. To further reveal carapace microbiota structure associated with ESD, we examined the composition of carapace microbiota at the genus level (Figure 3A). The number of bacterial genera that were found in any of the lobsters of each group was 367 in ESD and 465 in HTH, where 313 genera were shared in both groups. The number of bacterial genera that were found in any of the lobsters of each group with an average relative abundance of ≥0.1% was 64 in ESD and 81 in HTH, where 44 were shared between groups. Core genera refer to bacterial genera shared by all of the lobsters within each group. The number of core genera was 18 in ESD and 14 in HTH, where 4 were shared between groups. Core genera in ESD and HTH are listed in Supplementary Table S4. We also investigated the relative abundance of bacteria genera in the carapace of ESD and HTH. The top 10 most abundant bacterial genera in all samples included *Aquimarina, Halocynthiibacter, Tenacibaculum, Loktanella, Maritimimonas, Cocleimonas, Vibrio, Cohaesibacter, Maribacter,* and *Perspicuibacter* (Figure 3B). *Aquamarina* (13.5%), was the most abundant bacterial genus in ESD, and *Halocynthiibacter* (4.6%) was the most prevalent in HTH. There was no significant difference in the relative abundance of bacterial genera between ESD and HTH, except for *Aquimarina,* which was significantly higher (*p* < 0.01) in ESD than in HTH. To identify the function of carapace microbiota associated with ESD, we used PICRUSt, which predicts abundance of functional genes (i.e., KEGG) based on 16S rRNA gene data. The PICRUSt analysis of all carapace samples revealed the presence of 170 gene families in KEGG. Among them, 25 showed significant differences (*p* ≤ 0.05) between ESD and HTH (Figure 4), with 12 being more abundant in ESD and 13 more abundant in HTH. Notably, gene families involved in amino acid metabolism, including histidine, tyrosine, phenylalanine, and phosphonate and phosphinate metabolism, were enriched in ESD, whereas gene families involved in lipid metabolism, such as glycerophospholipid metabolism and primary bile acid biosynthesis, were enriched in HTH. Additionally, pathways related to genetic information processing, including base excision repair, sulfur relay system, aminoacyl-tRNA biosynthesis, and ribosome biogenesis in eukaryotes, were found to be more abundant in HTH than in ESD. Taken together, these data demonstrate that the structure and function of the carapace microbiota associated with ESD are distinct from those of healthy carapace microbiota.

### Presence of core carapace bacteria associated with epizootic shell disease in internal organs

**Figure 5:**
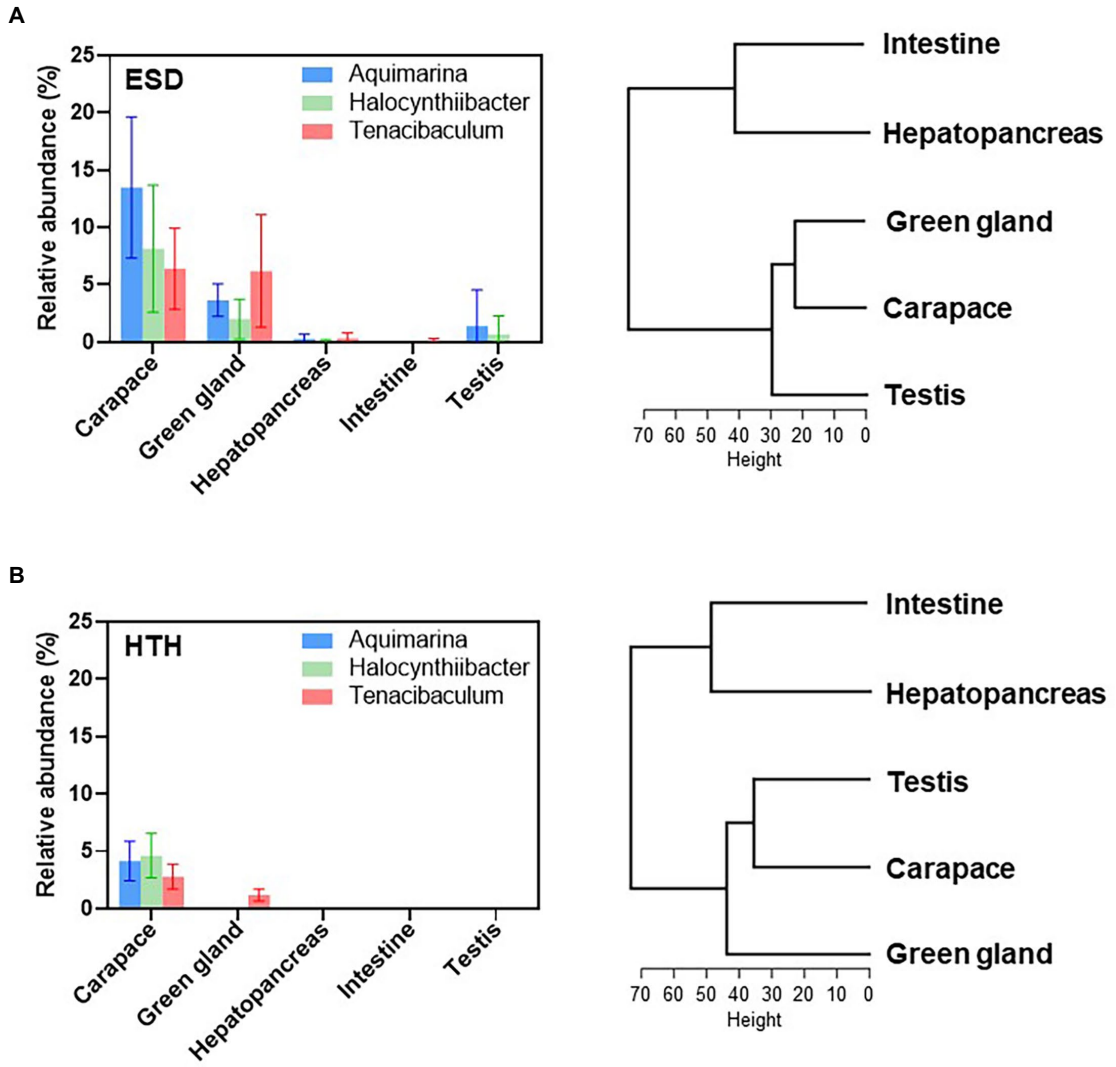
Effects of carapace bacteria on the internal tissues. Relative abundances of ESD-related core carapace bacteria (*Aquimarina*, *Halocynthiibacter,* and *Tenacibaculum*) across the carapace, green gland, hepatopancreas, intestine, and testis of **(A)** lobsters with ESD and **(B)** healthy lobsters. The bars represent the means and their standard errors. Hierarchical clustering analysis of five tissues based on the genus abundance data was conducted using the R function hclust.

To assess whether shell infection affects microbiota in the internal organs, we examined microbiota at the genus level in the green gland, hepatopancreas, intestine, and testis of both ESD and HTH. The lobsters showed different bacterial communities depending on their body site (Supplementary Figure S3). PCoA plots based on the Bray-Curtis distance showed no significant differences between ESD and HTH in the microbiota of the green gland, hepatopancreas, intestine, and testis (Supplementary Figure S4). We then investigated whether the three most abundant core carapace bacteria in ESD (*Aquimarina, Halocynthiibacter,* and *Tenacibaculum*) were present in the internal organs of lobsters. In lobsters with ESD (Figure 5A), *Aquimarina* and *Halocynthiibacter* were detected in the green gland, hepatopancreas, and testis, but not in the intestine. *Tenacibaculum* was present in the green gland, hepatopancreas, and intestine, except for the testis. The results of hierarchical clustering analysis, based on genus abundance data from five tissues, indicate that the carapace microbiota was more closely related to the green gland than other organs (Figure 5A). In contrast, in lobsters from HTH (Figure 5B), *Aquimarina* and *Halocynthiibacter* were absent in all internal organs tested, and *Tenacibaculum* was only detected in the green gland. Hierarchical clustering analysis revealed that the carapace microbiota was more similar to testis microbiota (Figure 5B). These findings suggest that carapace bacteria associated with ESD may have the ability to invade the internal tissues, particularly the green gland.

## Discussion

We investigated the carapace microbiota of healthy lobsters as well as lobsters with ESD to identify ESD-associated carapace bacteria. To avoid possible influence of geographic location on bacterial communities, lobsters were caught from three different regions (ELIS, WLIS, and offshore), but we were unable to obtain lobsters with ESD in the sample from WLIS. The absence or scarcity of ESD in WLIS is in accordance with previous studies, although the explanation remains unclear (Bell et al., 2012; Homerding et al., 2012). Lobsters caught from ELIS and offshore were therefore used in this study. The results of PCoA and PERMANOVA based on the Bray-Curtis distance show no significant difference (*p* = 0.11) in carapace microbiota between the two geographic locations (Supplementary Figure S5). Therefore, we focused our analysis on changes in carapace microbiota associated with health status.

American lobsters with ESD showed specific carapace microbiota characterized by high abundance of *Aquimarina*. Relative abundance of *Halocynthiibacter, Tenacibaculum,* and *Cohaesibacter* were also shown to be higher in ESD compared to HTH, but there were no significant differences. This may demonstrate that *Aquimarina* is the primary pathogen involved in ESD and others may act as secondary pathogens. The genus *Aquimarina* is a Gram-negative, aerobic, halophilic microorganism and affiliated with the phylum *Bacteroidota* (formerly *Bacteroidetes*) and the family *Flavobacteriaceae.* They are normally found in marine environments (Hahnke and Harder, 2013; Wang et al., 2016, 2018), as well as in various marine hosts such as algae, sponges, and lobsters (Kennedy et al., 2014; Kumar et al., 2016; Ranson et al., 2018). However, some *Aquimarina* species contain type 9 secretion system, gliding motility apparatus, and enzymes such as chitinase that breaks down crude chitin (Hudson et al., 2019; Silva et al., 2019), which may contribute to development of ESD in lobsters. Given that juvenile lobsters exposed to *Aquimarina* (*A. homaria* I32.4) did not develop ESD without artificial abrasion of the carapace (Quinn et al., 2012), *Aquimarina* could be an opportunistic pathogen. Further research is needed to elucidate the pathogenic mechanisms involved in the development of ESD.

The carapace microbiota of lobsters with ESD was found to contain several core bacterial genera, including *Halocynthiibacter, Tenacibaculum*, and *Cohaesibacter*. The genus *Halocynthiibacter*, which belongs to the phylum *Pseudomonadota* and the family *Rhodobacteraceae*, is a Gram-negative, aerobic, rod-shaped microorganism. It has been identified in sea squirt (Kim et al., 2014) and Artic marine sediment (Baek et al., 2015). The genus *Tenacibaculum* is a Gram-negative, aerobic, filamentous microorganism belonging to the phylum *Bacteroidota* and the family *Flavobacteriaceae*. *Tenacibaculum* species have been considered pathogenic bacteria responsible for mouth rot outbreaks in salmonid aquaculture (Frisch et al., 2018) and black-spot shell disease in pearl oysters (Sakatoku et al., 2018). The genus *Cohaesibacter* is a Gram-negative, facultative anaerobic, rod-shaped microorganism in the phylum *Pseudomonadota* and the family *Cohaesibacteraceae*. *Cohaesibacter* species have been isolated from coastal seawater and sediment (Hwang and Cho, 2008; Qu et al., 2011) as well as from the gut of sea catfish and abalone (Li et al., 2019; Liu et al., 2019). Recent studies have identified *Cohaesibacter* species as putative pathogens associated with stony coral tissue loss disease (Becker et al., 2022; Rosales et al., 2022). In our study, these core carapace bacteria were found to be more enriched in lobsters with ESD than healthy lobsters, although the differences were not significant. Further research is warranted if they play a role in the pathogenesis of ESD.

The bacterial communities of lobsters varied across their body sites. The green gland microbiota was dominated by *Vibrio* and *Arcobacter*, and the testis microbiota had a higher abundance of *Ensifer* and *Fusobacterium*. The hepatopancreas showed a microbiota dominated by *Candidatus Hepatoplasma* and *Photobacterium*, and the intestine revealed a microbiota dominated by *Photobacterium* and *Vibrio*. No significant differences were observed between lobsters with ESD and healthy lobsters, which could be attributed to the small sample size or the lack of impact of the disease on internal organ microbiota. Further investigation is needed to understand the effects of ESD on the bacterial communities in the internal organs of lobsters. It is noteworthy that the carapace microbiota was closely related to the green gland microbiota in lobsters with ESD, but in healthy lobsters, the carapace microbiota showed a closer association with the testis microbiota. This suggests that carapace bacteria associated with ESD may have a greater capacity to invade the green gland, highlighting the need for further investigation into the link between ESD and green gland function.

The carapace microbiota in lobsters with ESD was found to have an increased abundance of 12 gene families associated with various metabolic pathways, including four for amino acid metabolism, one for glycan biosynthesis and metabolism, two for carbohydrate metabolism, two for xenobiotics biodegradation and metabolism, one for metabolism of cofactors and vitamins, one for energy metabolism, and one for metabolism of terpenoids and polyketides. Under stressful environmental conditions, such as high temperatures and hypoxia, lobsters increase their energy metabolism to adapt to changing conditions (Podolski, 2011; Harrington et al., 2020). If the energy demand is not met, lobsters may become more susceptible to ESD (Tarrant et al., 2010). Nutrient deficiencies in lobsters can also affect the metabolisms of carapace bacteria, leading to increased energy production. The enrichment of amino acid metabolism pathways in carapace microbiota associated with ESD suggests that they may use amino acids as an energy source. On the other hand, healthy carapace microbiota was found to have an enrichment in lipid metabolisms, such as glycerophospholipid metabolism and primary bile acid biosynthesis. Given that carapace bacteria reside on the external surface of the shell, which has a lipid layer, it is reasonable to suggest that they may utilize lipids as an energy source (Tlusty et al., 2005; Bell et al., 2012). Our analysis of KEGG pathways suggests that the carapace microbiota of lobsters with ESD exhibits altered metabolic activity, which could be relevant to the pathogenesis of ESD.

We observed a significant difference in carapace microbiota structure between ESD and HTH using Illumina next-generation sequencing, which builds on previous studies that showed high abundance of *Aquimarina*, even though they were not able to discriminate bacterial communities associated with ESD from healthy ones (Bell et al., 2012; Chistoserdov et al., 2012; Meres et al., 2012). However, it should be noted that our study has some limitations. Our rarefaction curves demonstrate that we have achieved a sufficient sampling depth to accurately represent the diversity of carapace microbiota (Supplementary Figure S6). However, it is possible that additional species may exist in both the carapace and other body sites, which could be discovered through more extensive sampling efforts. We analyzed the data at either the OTU or genus levels, as it is challenging to accurately determine species-level taxonomy using Illumina short-read sequences. The lack of 16S rRNA gene sequence databases for marine environments further complicates species-level identification. Finally, the prediction of functional genes using PICRUSt may be less accurate in carapace samples compared to human samples because of a lack of bacterial reference genomes (Douglas et al., 2018; Sun et al., 2020). Nevertheless, this study provides valuable insights into the structure, diversity, and metabolic potential of carapace microbiota asscoated with ESD.

In conclusion, our study suggests that ESD may be associated with alterations in the structure and function of carapace microbiota. The genera *Aquimarina, Halocynthiibacter*, and *Tenacibaculum* were among the core bacteria associated with ESD, and they were also found in the green gland, possibly showing a subsequent internal infection. Further study is warranted to elucidate the roles of these carapace bacteria in the development of ESD.

## Data availability statement

BioSample metadata have been deposited in the NCBI BioSample database (http://www.ncbi.nlm.nih.gov/biosample/) under accession numbers SAMN33752320-SAMN33752359.

## Author contributions

SJ designed the study, interpreted the data, and revised the manuscript. AS performed the experiment and wrote the manuscript with the assistance of SJ. DC and VC collected lobster samples and revised the manuscript. SM analyzed the sequencing data and revised the manuscript. All authors contributed to the article and approved the submitted version.

## Funding

AS was funded by the Foundation for Food and Agriculture Research Vet Fellows Program for this research.

## Conflict of interest

The authors declare that the research was conducted in the absence of any commercial or financial relationships that could be construed as a potential conflict of interest.

## Publisher’s note

All claims expressed in this article are solely those of the authors and do not necessarily represent those of their affiliated organizations, or those of the publisher, the editors and the reviewers. Any product that may be evaluated in this article, or claim that may be made by its manufacturer, is not guaranteed or endorsed by the publisher.

## Footnotes

^1^ https://www.vandepeerlab.org/

## Supplementary material

SUPPLEMENTARY FIGURE S1 Alpha-diversity indices of carapace microbiota. **(A)** ACE. **(B)** Chao1. **(C)** Shannon. **(D)** Simpson. There were no significant differences in α-diversity indices between ESD and HTH (Mann-Whitney U test). Boxes represent the 25th and 75th percentiles with the median (lines inside the boxes). Error bars represent the 10th and 90th percentiles.

SUPPLEMENTARY FIGURE S2 PCoA plot based on the unweighted UniFrac distances of carapace microbiota. PERMANOVA showed no significant difference (*P* = 0.18) between ESD (red) and HTH (blue). The black circles represent the mean values, and ellipses indicate 95% confidence intervals.

SUPPLEMENTARY FIGURE S3 Microbiota by body site. **(A)** Carapace. **(B)** Green gland. **(C)** Hepatopancreas. **(D)** Intestine. **(E)** Testis. The relative abundance of the top 10 most abundant bacterial genera within each body site are plotted in stacked bar charts (Mann-Whitney U test, **P* ≤ 0.05).

SUPPLEMENTARY FIGURE S4 Bray-Curtis PCoA plots showing microbiota from the **(A)** green gland, **(B)** hepatopancreas, **(C)** intestine, and **(D)** testis. There were no significant differences between ESD (red) and HTH (blue) by one-way PERMANOVA..

SUPPLEMENTARY FIGURE S5 Bray-Curtis PCoA plot of carapace microbiota by health status (ESE vs. HTH) and geographical location (ELIS vs. Offshore) based on the genus abundance data. Two-way PERMANOVA revealed that carapace microbiota structure was significantly different by health status (*P* = 0.02), but not location (*P* = 0.11). Ellipses indicate 95% confidence intervals.

SUPPLEMENTARY FIGURE S6 Rarefaction curves of observed OTUs. **(A)** Rarefaction curves of all samples. **(B)** Rarefaction curves by body site (mean ± standard deviation).

SUPPLEMENTARY TABLE S1 Information on the animals and sampling site.

SUPPLEMENTARY TABLE S2 Sequencing data statistics and quality.

SUPPLEMENTARY TABLE S3 Relative abundance of carapace microbiota at each taxonomic level. Relative abundance of bacterial taxa was compared between ESD and HTH using Mann-Whitney U test.

SUPPLEMENTARY TABLE S4 Core bacterial genera in ESD and HTH.

## References

[ref1] Baek K. Lee Y. M. Shin S. C. Hwang K. Hwang C. Y. Hong S. G.. (2015). Halocynthiibacter arcticus sp. nov., isolated from Arctic marine sediment. Int. J. Syst. Evol. Microbiol. 65, 3861–3865. doi: 10.1099/ijsem.0.000507, PMID: 26243211

[ref2] Becker C. C. Brandt M. Miller C. A. Apprill A. (2022). Microbial bioindicators of stony coral tissue loss disease identified in corals and overlying waters using a rapid field-based sequencing approach. Environ. Microbiol. 24, 1166–1182. doi: 10.1111/1462-2920.15718, PMID: 34431191

[ref3] Bell S. L. Allam B. McElroy A. Dove A. Taylor G. T. (2012). Investigation of epizootic shell disease in American lobsters (*Homarus americanus*) from Long Island sound: I characterization of associated microbial communities. J. Shellfish Res. 31, 473–484. doi: 10.2983/035.031.0207

[ref4] Bolyen E. Rideout J. R. Dillon M. R. Bokulich N. A. Abnet C. C. al-Ghalith G. A.. (2019). Reproducible, interactive, scalable and extensible microbiome data science using QIIME 2. Nat. Biotechnol. 37, 852–857. doi: 10.1038/s41587-019-0209-9, PMID: 31341288 PMC7015180

[ref5] Castro K. M. Cobb J. S. Gomez-Chiarri M. Tlusty M. (2012). Epizootic shell disease in American lobsters *Homarus americanus* in southern New England: past, present and future. Dis. Aquat. Org. 100, 149–158. doi: 10.3354/dao02507, PMID: 23186702

[ref6] Chistoserdov A. Y. Quinn R. A. Gubbala S. L. Smolowitz R. (2012). Bacterial communities associated with lesions of shell disease in the American lobster *Homarus americanus* Milne-Edwards. J Shellfish Res 31, 449–462. doi: 10.2983/035.031.0205

[ref8] Douglas G. M. Beiko R. G. Langille M. G. I. (2018). Predicting the functional potential of the microbiome from marker genes using PICRUSt. Methods Mol. Biol. 1849, 169–177. doi: 10.1007/978-1-4939-8728-3_11, PMID: 30298254

[ref9] Frisch K. Småge S. B. Vallestad C. Duesund H. Brevik O. J. Klevan A.. (2018). Experimental induction of mouthrot in Atlantic salmon smolts using Tenacibaculum maritimum from Western Canada. J. Fish Dis. 41, 1247–1258. doi: 10.1111/jfd.12818, PMID: 29761493

[ref10] Goldstein J. S. Zarrella-Smith K. A. Pugh T. L. (2022). Recent declines in American lobster fecundity in southern New England: drivers and implications. ICES J. Mar. Sci. 79, 1662–1674. doi: 10.1093/icesjms/fsac083

[ref11] Gregory R Warnes B Lodewijk B. (2021). Gplots: various R programming tools for plotting data R package version 3.

[ref12] Groner M. L. Shields J. D. Landers D. F. Jr. Swenarton J. Hoenig J. M. (2018). Rising temperatures, molting phenology, and epizootic shell disease in the American lobster. Am. Nat. 192, E163–E177. doi: 10.1086/699478, PMID: 30332587

[ref13] Hahnke R. L. Harder J. (2013). Phylogenetic diversity of Flavobacteria isolated from the North Sea on solid media. Syst. Appl. Microbiol. 36, 497–504. doi: 10.1016/j.syapm.2013.06.006, PMID: 23957959

[ref14] Hammer Ø. Harper D. A. Ryan P. D. (2001). PAST: paleontological statistics software package for education and data analysis. Palaeontol. Electron. 4:9. Available online at: https://paleo.carleton.ca/2001_1/past/past.pdf

[ref15] Harrington A. M. Clark K. F. Hamlin H. J. (2020). Expected Ocean warming conditions significantly alter the transcriptome of developing postlarval American lobsters (Homarus *americanus*): implications for energetic trade-offs. Comp. Biochem. Phys. D. 36:100716. doi: 10.1016/j.cbd.2020.100716 32777773

[ref16] Harvell C. D. Mitchell C. E. Ward J. R. Altizer S. Dobson A. P. Ostfeld R. S.. (2002). Climate warming and disease risks for terrestrial and marine biota. Science 296, 2158–2162. doi: 10.1126/science.1063699, PMID: 12077394

[ref17] Homerding M. Mcelroy A. Taylor G. Dove A. Allam B. (2012). Investigation of epizootic shell disease in American lobsters (*Homarus americanus*) from Long Island sound: II. Immune parameters in lobsters and relationships to the disease. J. Shellfish Res. 31, 495–504. doi: 10.2983/035.031.0209

[ref18] Howell P. (2012). The status of the southern New England lobster stock. J. Shellfish Res. 31, 573–579. doi: 10.2983/035.031.0217

[ref19] Howell P. Benway J. Giannini C. McKown K. Burgess R. Hayden J. (2005). Long-term population trends in American lobster (*Homarus americanus*) and their relation to temperature in Long Island sound. J. Shellfish Res. 24, 849–857. doi: 10.2983/0730-8000(2005)24[849:LPTIAL]2.0.CO;2

[ref20] Hudson J. Kumar V. Egan S. (2019). Comparative genome analysis provides novel insight into the interaction of Aquimarina sp. AD1, BL5 and AD10 with their macroalgal host. Mar. Genom. 46, 8–15. doi: 10.1016/j.margen.2019.02.005, PMID: 30852185

[ref21] Hwang C. Y. Cho B. C. (2008). Cohaesibacter gelatinilyticus gen. Nov., sp. nov., a marine bacterium that forms a distinct branch in the order Rhizobiales, and proposal of Cohaesibacteraceae fam. Nov. Int. J. Syst. Evol. Microbiol. 58, 267–277. doi: 10.1099/ijs.0.65016-0 18175720

[ref22] Kennedy J. Margassery L. M. O'Leary N. D. O'Gara F. Morrissey J. Dobson A. D. W. (2014). Aquimarina amphilecti sp. nov., isolated from the sponge Amphilectus fucorum. Int. J. Syst. Evol. Microbiol. 64, 501–505. doi: 10.1099/ijs.0.049650-0, PMID: 24108324

[ref23] Kim Y. O. Park S. Kim H. Park D. S. Nam B. H. Kim D. G.. (2014). Halocynthiibacter namhaensis gen. Nov., sp. nov., a novel alphaproteobacterium isolated from sea squirt Halocynthia roretzi. Antonie Van Leeuwenhoek 105, 881–889. doi: 10.1007/s10482-014-0142-3, PMID: 24573327

[ref24] Kumar V. Zozaya-Valdes E. Kjelleberg S. Thomas T. Egan S. (2016). Multiple opportunistic pathogens can cause a bleaching disease in the red seaweed Delisea pulchra. Environ. Microbiol. 18, 3962–3975. doi: 10.1111/1462-2920.13403, PMID: 27337296

[ref26] Langille M. G. Zaneveld J. Caporaso J. G. McDonald D. Knights D. Reyes J. A.. (2013). Predictive functional profiling of microbial communities using 16S rRNA marker gene sequences. Nat. Biotechnol. 31, 814–821. doi: 10.1038/nbt.2676, PMID: 23975157 PMC3819121

[ref27] Li Y. X. Wang N. N. Chen G. J. Du Z. J. (2019). Cohaesibacter celericrescens sp. nov., isolated from sea catfish. Int. J. Syst. Evol. Microbiol. 69, 255–260. doi: 10.1099/ijsem.0.003146, PMID: 30489240

[ref28] Liu M. Huang Z. Zhao Q. Shao Z. (2019). Cohaesibacter intestini sp. nov., isolated from the intestine of abalone, Haliotis discus hannai. Int. J. Syst. Evol. Microbiol. 69, 3202–3206. doi: 10.1099/ijsem.0.003610, PMID: 31339485

[ref29] Long Island Sound Study. Lobster Abundance. Available online at: https://longislandsoundstudy.net/ecosystem-target-indicators/lobster-abundance/.

[ref30] Meres N. J. Ajuzie C. C. Sikaroodi M. Vemulapalli M. Shields J. D. Gillevet P. M. (2012). Dysbiosis in epizootic shell disease of the American lobster (*Homarus americanus*). J. Shellfish Res. 31, 463–472. doi: 10.2983/035.031.0206

[ref25] National Oceanic and Atmospheric Administration. Landings. Available online at: https://www.fisheries.noaa.gov/foss/f?p=215:200:13299770287257::NO:RP.

[ref7] National Oceanic and Atmospheric Administration (2016). Climate & Lobsters. Available online at: https://www.climate.gov/news-features/climate-and/climate-lobsters.

[ref31] Parks D. H. Tyson G. W. Hugenholtz P. Beiko R. G. (2014). STAMP: statistical analysis of taxonomic and functional profiles. Bioinformatics 30, 3123–3124. doi: 10.1093/bioinformatics/btu494, PMID: 25061070 PMC4609014

[ref32] Podolski S. (2011). Activation of AMP-activated protein kinase as an early indicator for stress in the lobster, *Homarus americanus.* (Maine: University of New England). Available at: https://dune.une.edu/cgi/viewcontent.cgi?referer=&httpsredir=1&article=1001&context=theses

[ref33] Qu L. Y. Lai Q. L. Zhu F. L. Hong X. G. Sun X. Q. Shao Z. Z. (2011). Cohaesibacter marisflavi sp. nov., isolated from sediment of a seawater pond used for sea cucumber culture, and emended description of the genus Cohaesibacter. Int. J. Syst. Evol. Micr. 61, 762–766. doi: 10.1099/ijs.0.021972-0, PMID: 20418408

[ref34] Quinn B. K. (2017). Threshold temperatures for performance and survival of American lobster larvae: a review of current knowledge and implications to modeling impacts of climate change. Fish. Res. 186, 383–396. doi: 10.1016/j.fishres.2016.09.022

[ref35] Quinn R. A. Metzler A. Smolowitz R. M. Tlusty M. Chistoserdov A. Y. (2012). Exposures of *Homarus americanus* shell to three bacteria isolated from naturally occurring epizootic shell disease lesions. J. Shellfish Res. 31, 485–493. doi: 10.2983/035.031.0208

[ref36] Ranson H. J. LaPorte J. Spinard E. Chistoserdov A. Y. Gomez-Chiarri M. Nelson D. R.. (2018). Draft genome sequence of the putative marine pathogen Aquimarina sp. strain I32.4. Genome Announc. 6, e00313–e00318. doi: 10.1128/genomeA.00313-18 29700150 PMC5920181

[ref37] Rognes T. Flouri T. Nichols B. Quince C. Mahe F. (2016). VSEARCH: a versatile open source tool for metagenomics. PeerJ 4:e2584. doi: 10.7717/peerj.2584, PMID: 27781170 PMC5075697

[ref38] Rosales S. M. Huebner L. K. Clark A. S. McMinds R. Ruzicka R. R. Muller E. M. (2022). Bacterial metabolic potential and micro-eukaryotes enriched in stony coral tissue loss disease lesions. Front. Mar. Sci. 8:776859. doi: 10.3389/fmars.2021.776859

[ref39] Sakatoku A. Fujimura T. Ito M. Takashima S. Isshiki T. (2018). Newly isolated bacterium Tenacibaculum sp strain Pbs-1 from diseased pearl oysters is associated with black-spot shell disease. Aquaculture 493, 61–67. doi: 10.1016/j.aquaculture.2018.04.049

[ref40] Shields J. D. (2013). Complex etiologies of emerging diseases in lobsters (*Homarus americanus*) from Long Island sound. Can. J. Fish. Aquat. Sci. 70, 1576–1587. doi: 10.1139/cjfas-2013-0050

[ref41] Shields J. D. (2019). Climate change enhances disease processes in crustaceans: case studies in lobsters, crabs, and shrimps. J. Crustac. Biol. 39, 673–683. doi: 10.1093/jcbiol/ruz072

[ref42] Silva S. G. Blom J. Keller-Costa T. Costa R. (2019). Comparative genomics reveals complex natural product biosynthesis capacities and carbon metabolism across host-associated and free-living Aquimarina (Bacteroidetes, Flavobacteriaceae) species. Environ. Microbiol. 21, 4002–4019. doi: 10.1111/1462-2920.14747, PMID: 31314938

[ref43] Smolowitz R. Chistoserdov A. Hsu A. (2005). “Epizootic shell disease in the American lobster, *Homarus americanus*” in State of lobster science: Lobster shell disease workshop 2005 mar 12 (Boston, MA: New England Aquarium), 2–11.

[ref44] Sun S. Jones R. B. Fodor A. A. (2020). Inference-based accuracy of metagenome prediction tools varies across sample types and functional categories. Microbiome 8, 1–9. doi: 10.1186/s40168-020-00815-y 32241293 PMC7118876

[ref45] Tarrant A. M. Stegeman J. J. Verslycke T. (2010). Altered gene expression associated with epizootic shell disease in the American lobster *Homarus americanus*. Fish Shellfish Immunol. 29, 1003–1009. doi: 10.1016/j.fsi.2010.08.008, PMID: 20728539

[ref46] Tlusty MF Halvorson HO Smolowitz R Sharma U. (2005). State of lobster science: Shell disease workshop. Aquatic Forum Series 05-1. The New England Aquarium, Boston, Massachusetts.

[ref47] Tlusty M. F. Metzler A. (2012). Relationship between temperature and shell disease in laboratory populations of juvenile American lobsters (*Homarus americanus*). J. Shellfish Res. 31, 533–541. doi: 10.2983/035.031.0213

[ref48] Wang Y. Ming H. Guo W. Chen H. Zhou C. (2016). Aquimarina aggregata sp. nov., isolated from seawater. Int. J. Syst. Evol. Microbiol. 66, 3406–3412. doi: 10.1099/ijsem.0.001209, PMID: 27259860

[ref49] Wang N. N. Zhou L. Y. Li Y. X. Du Z. J. (2018). Aquimarina sediminis sp. nov., isolated from coastal sediment. Antonie Van Leeuwenhoek 111, 2257–2265. doi: 10.1007/s10482-018-1115-8, PMID: 29915892

[ref50] Wickham H. (2016). ggplot2: Elegant graphics for data analysis R package version. Cham: Springer-Verlag New York.

