# Supplementary figures and images for: Carapace microbiota in American lobsters (Homarus americanus) associated with epizootic shell disease and the green gland
Source: Front Microbiol. 2023 Apr 5;14:1093312. doi: 10.3389/fmicb.2023.1093312 (PMC10113626; doi:10.3389/fmicb.2023.1093312)

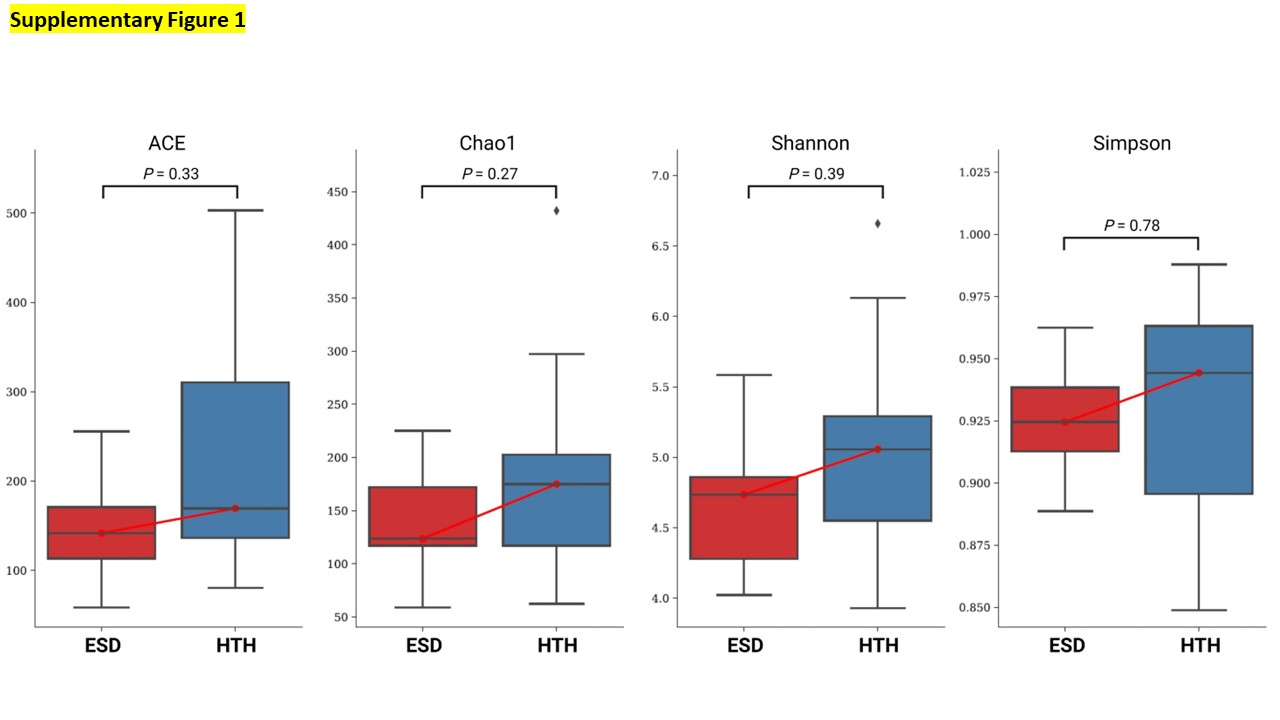

Supplement: SUPPLEMENTARY FIGURE S1 — Alpha-diversity indices of carapace microbiota. (A) ACE. (B) Chao1. (C) Shannon. (D) Simpson. There were no significant differences in α-diversity indices between ESD and HTH (Mann-Whitney U test). Boxes represent the 25th and 75th percentiles with the median (lines inside the boxes). Error bars represent the 10th and 90th percentiles. [file Image_1.JPEG]

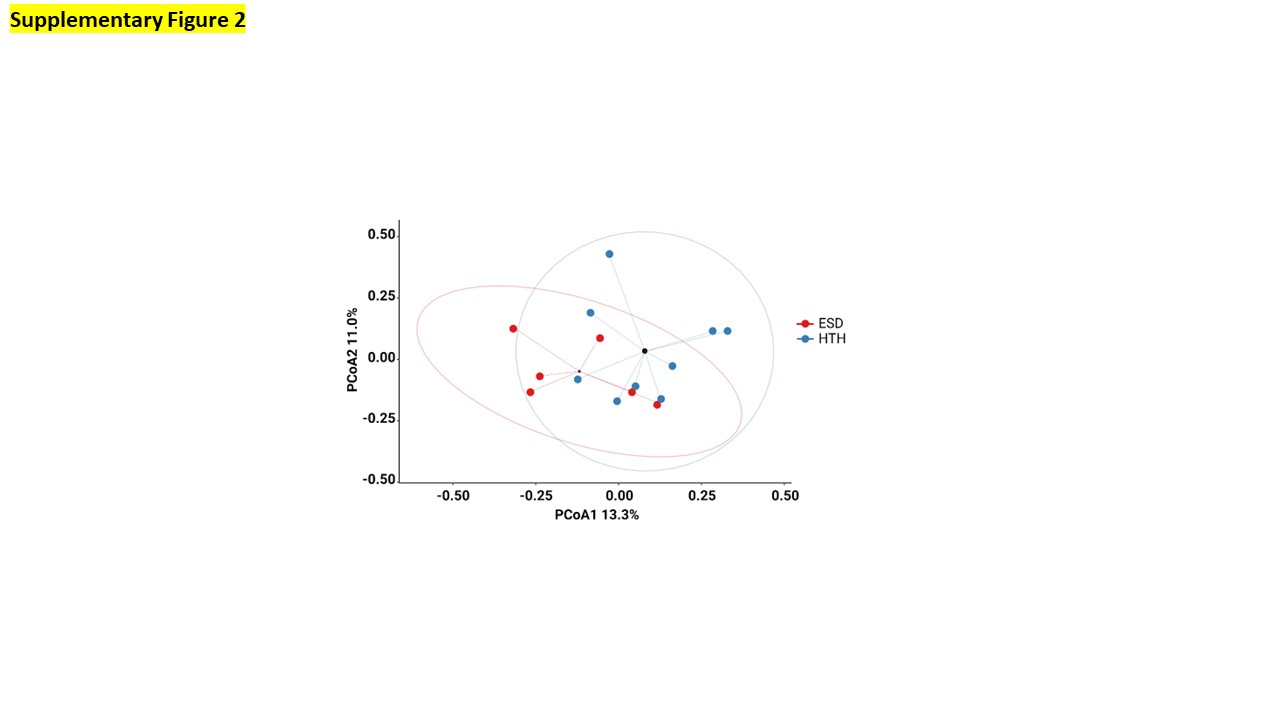

Supplement: SUPPLEMENTARY FIGURE S2 — PCoA plot based on the unweighted UniFrac distances of carapace microbiota. PERMANOVA showed no significant difference (P = 0.18) between ESD (red) and HTH (blue). The black circles represent the mean values, and ellipses indicate 95% confidence intervals. [file Image_2.JPEG]

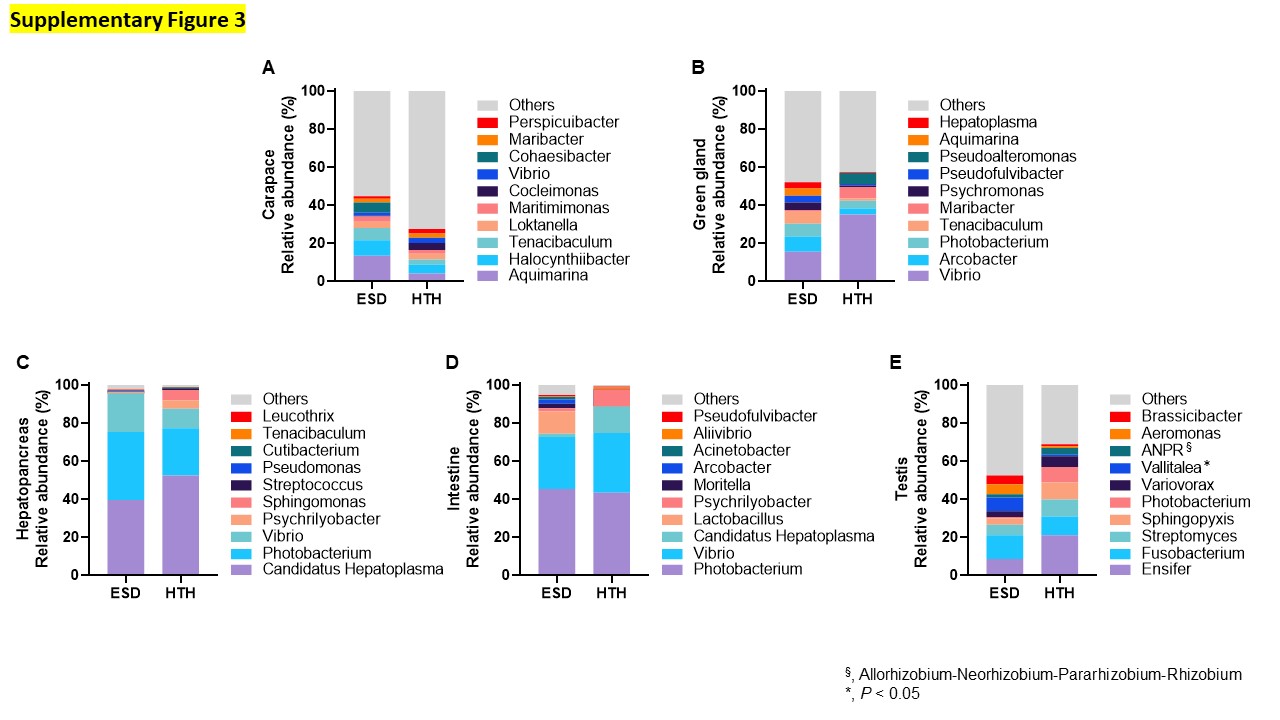

Supplement: SUPPLEMENTARY FIGURE S3 — Microbiota by body site. (A) Carapace. (B) Green gland. (C) Hepatopancreas. (D) Intestine. (E) Testis. The relative abundance of the top 10 most abundant bacterial genera within each body site are plotted in stacked bar charts (Mann-Whitney U test, *P ≤ 0.05). [file Image_3.JPEG]

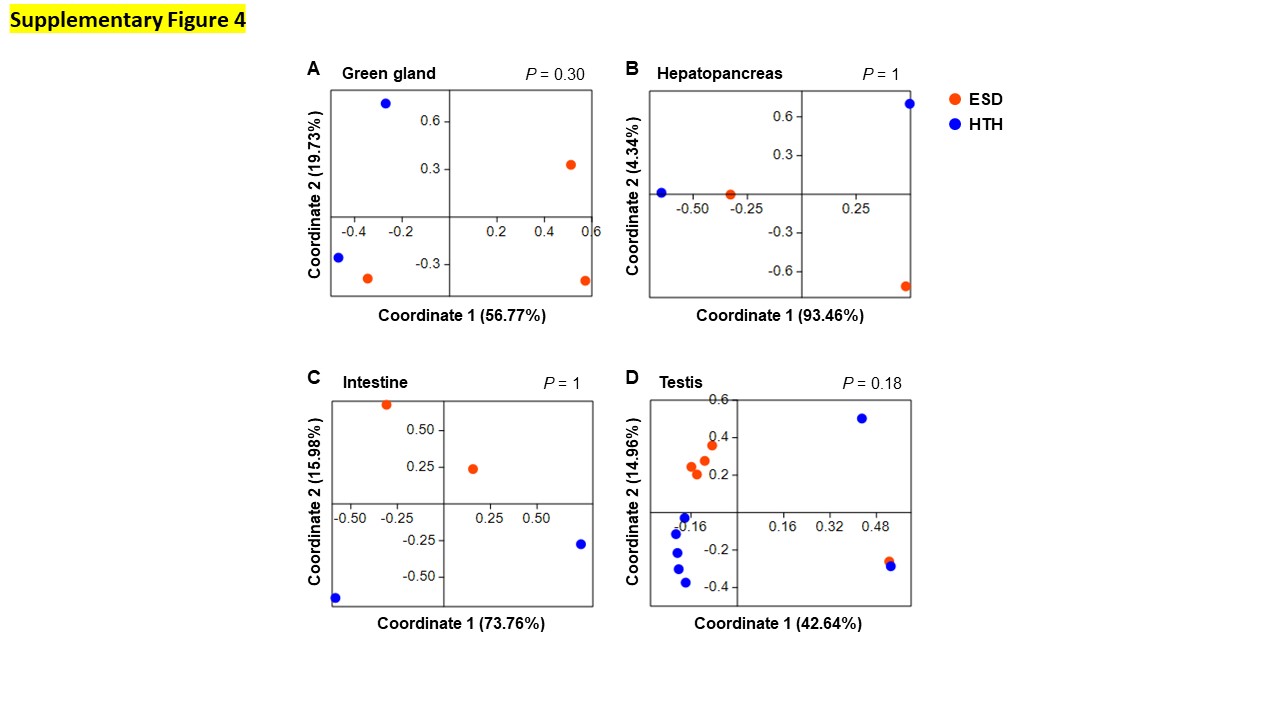

Supplement: SUPPLEMENTARY FIGURE S4 — Bray-Curtis PCoA plots showing microbiota from the (A) green gland, (B) hepatopancreas, (C) intestine, and (D) testis. There were no significant differences between ESD (red) and HTH (blue) by one-way PERMANOVA.. [file Image_4.JPEG]

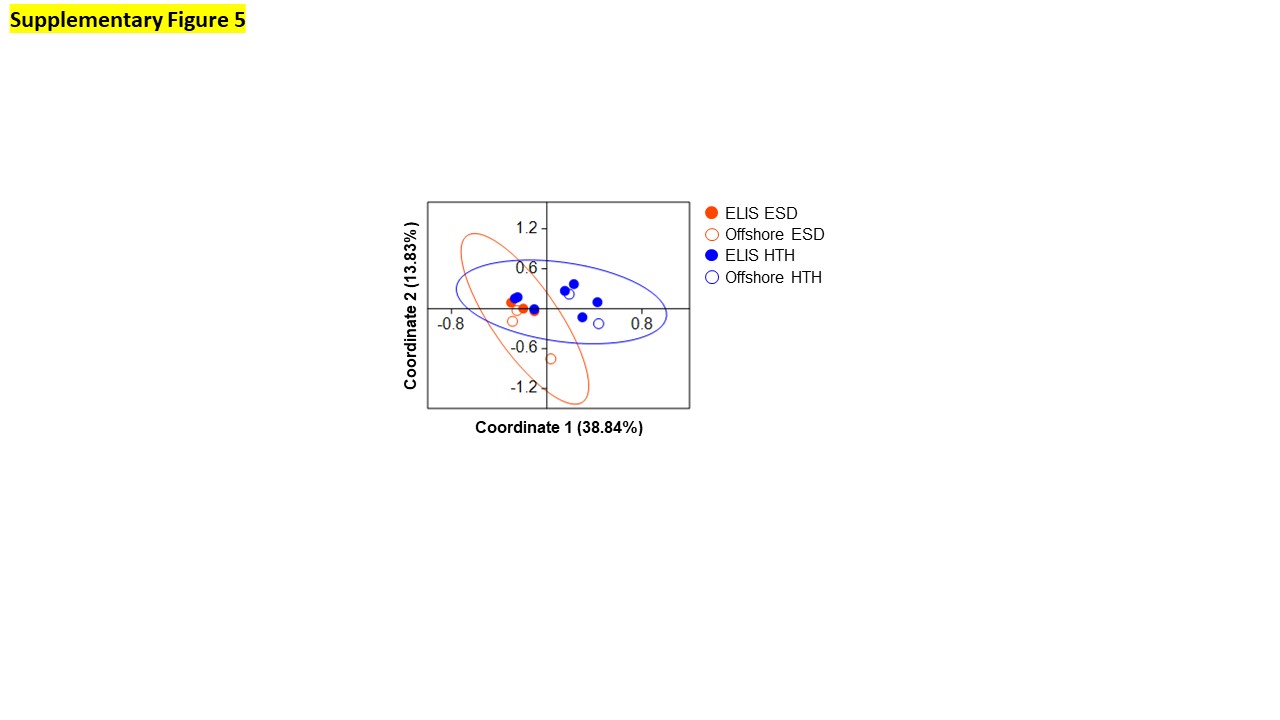

Supplement: SUPPLEMENTARY FIGURE S5 — Bray-Curtis PCoA plot of carapace microbiota by health status (ESE vs. HTH) and geographical location (ELIS vs. Offshore) based on the genus abundance data. Two-way PERMANOVA revealed that carapace microbiota structure was significantly different by health status (P = 0.02), but not location (P = 0.11). Ellipses indicate 95% confidence intervals. [file Image_5.JPEG]

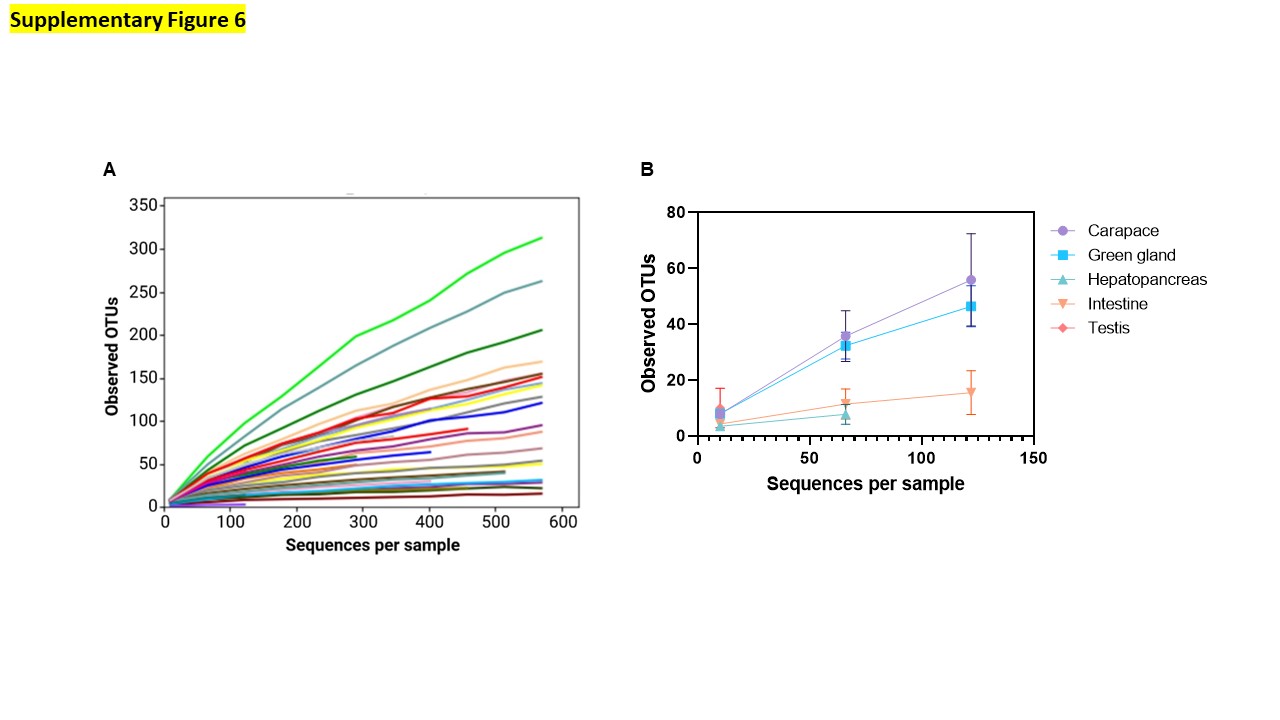

Supplement: SUPPLEMENTARY FIGURE S6 — Rarefaction curves of observed OTUs. (A) Rarefaction curves of all samples. (B) Rarefaction curves by body site (mean ± standard deviation). [file Image_6.JPEG]
